# Suppressive Effects of Bee Venom-Derived Phospholipase A2 on Mechanical Allodynia in a Rat Model of Neuropathic Pain

**DOI:** 10.3390/toxins11080477

**Published:** 2019-08-19

**Authors:** Seunghui Woo, Geehoon Chung, Hyunsu Bae, Sun Kwang Kim

**Affiliations:** 1 Department of Science in Korean Medicine, Graduate School, Kyung Hee University, Seoul 02447, Korea; 2 Department of Physiology, College of Korean Medicine, Kyung Hee University, Seoul 02447, Korea

**Keywords:** phospholipase A2, bee venom, peripheral nerve injury, neuropathic pain, mechanical allodynia, spinal cord, adrenergic receptor

## Abstract

Bee venom (BV) has a long history of being used in traditional Korean medicine to relieve pain. Here, we investigated the effect of BV-derived phospholipase A2 (bvPLA2), a major component of BV, on peripheral nerve injury-induced neuropathic pain in rats. Spinal nerve ligation (SNL) was performed in Sprague Dawley rats to induce neuropathic pain, and paw withdrawal thresholds were measured using von Frey test. Mechanical allodynia, the representative symptom of neuropathic pain, was manifested following SNL and persisted for several weeks. The repetitive bvPLA2 treatment (0.2 mg/kg/day, i.p.) for two days significantly relieved the SNL-induced mechanical allodynia. The antiallodynic effect of bvPLA2 was blocked by spinal pretreatment with α1-adrenergic antagonist prazosin (30 μg, i.t.) but not with α2-adrenergic antagonist idazoxan (50 μg, i.t.). Also, the spinal application of α1-adrenergic agonist phenylephrine (50 μg, i.t.) reduced mechanical allodynia. These results indicate that bvPLA2 could relieve nerve injury-induced neuropathic mechanical allodynia through the activation of spinal α1-adrenergic receptors.

## 1. Introduction

Neuropathic pain is normally defined as pain caused by nerve damage or dysfunction of the somatosensory system and, unlike nociceptive pain, has no warning function useful for survival. Neuropathic pain patients experience symptoms of allodynia, hyperalgesia, and painful sensation like shooting, burning, and tingling. In the clinic, anticonvulsants (e.g., gabapentin, pregabalin) and antidepressants (e.g., duloxetine) are currently being used for neuropathic pain patients. However, these treatments are only effective in a subset of patients, and there are adverse effects that restrict the use of the treatments for patients [1,2]. Hence, it is necessary to discover new alternative therapies.

Bee venom (BV) is naturally secreted by bees via their sting and has long been used to alleviate pain and to treat chronic inflammatory diseases in Korean medicine [3]. In particular, the analgesic effect of BV has been studied in various pain states, including formalin-induced pain, chemotherapy-induced peripheral neuropathy (CIPN), and nerve injury-induced neuropathic pain [4,5,6]. These studies also indicated the crucial role of noradrenergic system in analgesic effect of BV [5,6]. 

BV consists of various bioactive molecules, including enzymes and peptides. Phospholipase A2 derived from BV (bvPLA2) is a main component of BV, occupying 12% of dry BV. The early studies had reported that bvPLA2 is a major allergen that can induce anaphylaxis [7]. On the contrary, recent studies have demonstrated that bvPLA2 modulates regulatory T cells (Treg cells) and induces beneficial effects, including suppression of autoimmune symptoms [8,9,10]. Furthermore, increasing evidence suggests that bvPLA2 has many therapeutic effects, including anti-neurodegenerative, anti-inflammatory, and anticancer effects [11,12,13]. In addition, our previous studies have shown that bvPLA2 treatment is effective in preventing and relieving neuropathic pain in CIPN models. Consecutive injection of bvPLA2 for five days prior to oxaliplatin injection could prevent the development of mechanical and cold allodynia [14], and subcutaneous injection of bvPLA2 was found to attenuate paclitaxel-induced mechanical allodynia in rats [5]. Furthermore, we found that repetitive bvPLA2 treatments could attenuate oxaliplatin-induced neuropathic pain symptoms, including cold and mechanical allodynia, and the analgesic effects were mediated by the noradrenergic system but not by the serotonergic system [15]. Noradrenergic projection starting from the midbrain area plays a crucial role in pain modulation by regulating the spinal neuronal excitability. Impairment of this inhibitory pathway is related to induction and maintenance of neuropathic pain, and activating this noradrenergic pathway is considered as an important strategy to treat neuropathic pain [16,17]. 

Although these previous studies identified the analgesic effects of bvPLA2 treatments on neuropathic pain condition, the studies used CIPN models of which the pathological mechanisms are slightly different from neuropathic pain induced by physical nerve injury [18]. The symptoms of CIPN diminishes after the cessation of the chemotherapeutic agent, whereas the symptoms of nerve injury-induced neuropathic pain persist for several months [19]. Here, we investigated whether repetitive treatment with bvPLA2 could relieve peripheral nerve injury-induced sustained allodynia. Then, we determined whether the effects of the bvPLA2 treatment were mediated by activation of the spinal noradrenergic system. 

## 2. Results

### 2.1. L5 Spinal Nerve Ligation Induces Mechanical Allodynia in the Affected Hind Paw

**Figure 1:**
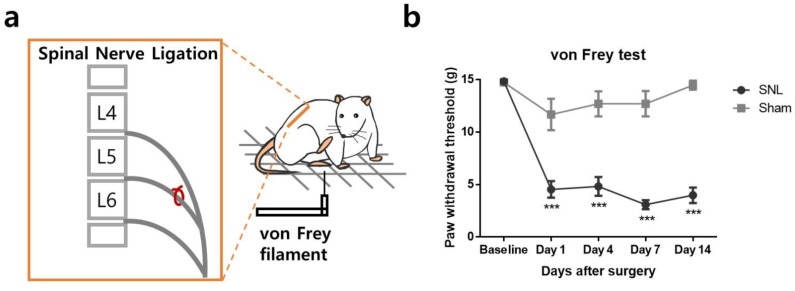
Assessment of mechanical allodynia following the right L5 spinal nerve ligation (SNL) surgery. (**a**) Scheme of the experimental procedure. The right L5 spinal nerve was carefully isolated and ligated (**left**). Paw withdrawal threshold of the right hind paw was evaluated using von Frey filaments with different bending forces (**right**). (**b**) After the surgery, SNL rats (*n* = 18) showed reduced paw withdrawal threshold compared to sham rats (*n* = 11). The data are presented as mean ± standard error of mean (SEM). (*** *p* < 0.001; two-way ANOVA followed by Bonferroni’s multiple comparison test).

Spinal nerve ligation (SNL) surgery was carried out in male Sprague Dawley rats to induce neuropathic mechanical allodynia. Sham surgery was performed in the control group. We measured the behavioral sign of mechanical allodynia before and 1, 4, 7, 14 days after the surgery using von Frey filaments. As shown in Figure 1, mechanical allodynia was successfully induced in the SNL group, indicated by a significant reduction of paw withdrawal threshold (PWT) in the ipsilateral hind paw compared to the sham group. 

### 2.2. Repetitive bvPLA2 Treatments Markedly Attenuate Mechanical Allodynia in SNL Rats

**Figure 2:**
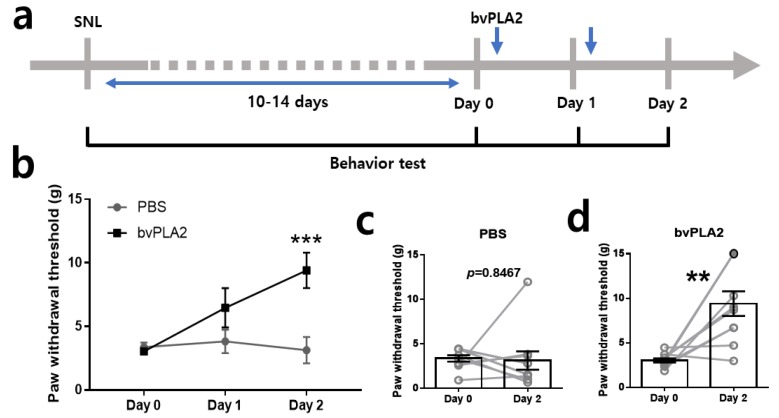
Reduction of mechanical allodynia after two repetitive injections of bee venom-derived phospholipase A2 (bvPLA2). (**a**) Experimental schedule of bvPLA2 treatment and behavior test. (**b**) Time course of the analgesic effect of bvPLA2 on mechanical allodynia in SNL rats (*** *p* < 0.001; two-way ANOVA followed by Bonferroni’s multiple comparison test). Comparison of paw withdrawal thresholds (PWTs) before and after the repetitive injections of (**c**) phosphate-buffered saline (PBS) and (**d**) bvPLA2 (*n* = 10 for each group, ** *p* < 0.01; paired *t*-test). All data are expressed as mean ± SEM.

We investigated whether bvPLA2 reduces SNL-induced mechanical allodynia. Treatment with bvPLA2 (0.2 mg/kg, i.p.) or vehicle (phosphate-buffered saline, PBS) was started between 10 and 14 days after the SNL surgery (Figure 2a). The paw withdrawal threshold was measured 24 h after each injection in these behavioral tests. In bvPLA2-injected SNL group, mechanical allodynia was gradually reversed compared to the PBS-treated SNL group (Figure 2b). A significant difference was observed after two repetitive injections but not after the first injection (Figure 2b). Individual differences following two repetitive injections are represented in Figure 2c,d. Almost all the PBS-treated SNL rats showed no change (Figure 2c). Interestingly, SNL-induced mechanical allodynia was completely diminished in 30% of the bvPLA2-treated SNL group (three out of 10 rats, filled circle) after two repetitive bvPLA2 injections (Figure 2d).

### 2.3. α1-Adrenergic Receptor (AR) Is Involved in the Antiallodynic Effects of bvPLA2

**Figure 3:**
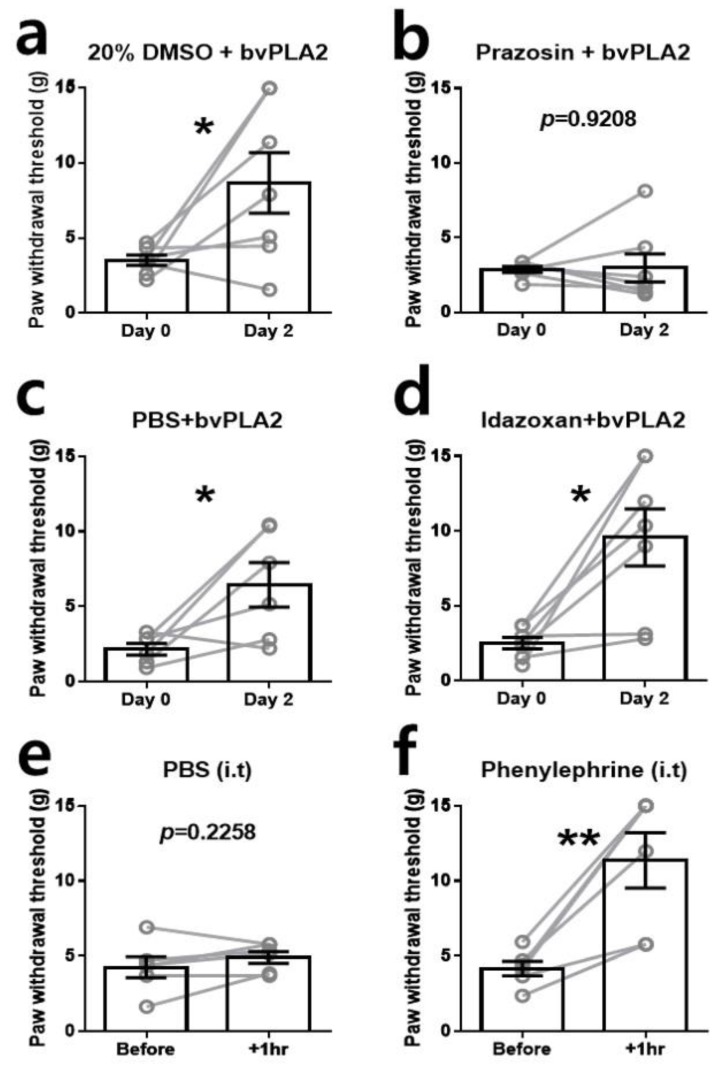
Involvement of α1-adrenergic receptor (AR) in the analgesic effect of bvPLA2. Antagonists and its vehicles were intrathecally administered 20 min before the intraperitoneal injection of bvPLA2 (**a**–**d**). For antagonist test, rats were divided into four groups. (**a**) Control group for prazosin pretreatment. Vehicle for prazosin (dimethyl sulfoxide (DMSO) 20%) was used prior to bvPLA2 treatment (*n* = 7). (**b**) Prazosin pretreatment group. The α1-AR antagonist prazosin completely blocked the analgesic effect of bvPLA2 treatment (*n* = 7). (**c**) Control group for idazoxan pretreatment. Vehicle for idazoxan (PBS solution) was used for pretreatment (*n* = 6). (**d**) Idazoxan pretreatment group. The α2-AR antagonist idazoxan could not block the bvPLA2-mediated analgesic effect (*n* = 7). (**e**,**f**) The analgesic effect induced by intrathecal injection of α1-AR agonist phenylephrine. Direct activation of spinal α1-AR by intrathecal phenylephrine injection ameliorated SNL-induced mechanical allodynia one hour after the treatment (*n* = 6 for each group). The data are expressed as mean ± SEM. (* *p* < 0.05, ** *p* < 0.01; paired *t*-test).

Previous studies have shown that analgesic effects of BV or bvPLA2 are mediated by activation of spinal noradrenergic system, which mainly involves α-ARs. In the same way, we investigated whether α-ARs play a role in the analgesic effect of bvPLA2 on SNL-induced mechanical allodynia. Prazosin (α_1_-AR antagonist, 30 μg/kg) or idazoxan (α_2_-AR antagonist, 50 μg/kg) was intrathecally administered before the intraperitoneal treatment with bvPLA2. Vehicle solutions were used as controls for each antagonist (dimethyl sulfoxide (DMSO) for prazosin and PBS for idazoxan). We found that the pretreatment with prazosin, the α_1_-AR antagonist, blocked the analgesic effect of bvPLA2 treatment (Figure 3b). The pretreatment with idazoxan, the α_2_-AR antagonist, could not block the analgesic effect of bvPLA2 (Figure 3d). These results indicate that the analgesic effects of bvPLA2 in SNL-induced mechanical allodynia are mediated by activation of spinal α_1_- but not α_2_-ARs.

### 2.4. A Single Injection of α_1_-AR Agonist Ameliorates Mechanical Allodynia

To check whether α_1_-AR activation could mediate the analgesic effect in neuropathic pain condition, we assessed the mechanical allodynia in SNL rats one hour after intrathecal injection of phenylephrine (α_1_-AR agonist, 50 μg). Phenylephrine injection ameliorated SNL-induced mechanical allodynia, as shown by a significant increase in the paw withdrawal threshold (Figure 3e,f). This result indicates that the direct activation of the spinal α_1_-AR can induce the powerful analgesic effect in SNL-induced neuropathic pain condition. 

## 3. Discussion

bvPLA2 is a type of secretory PLA2 that has been widely used to treat chronic inflammatory and neurodegenerative diseases [13]. In this study, we aimed to investigate whether bvPLA2 treatment could be a possible alternative treatment for nerve injury-induced neuropathic pain. To achieve this goal, the analgesic effect of bvPLA2 treatment on SNL-induced mechanical allodynia was assessed. Firstly, we confirmed the successful induction of pain symptom in rats performed with L5 SNL surgery. The behavioral results were consistent with data from other studies that used the same animal model [20,21]. In subsequent experiments, we examined whether repetitive treatments with bvPLA2 had analgesic effects on SNL-induced mechanical allodynia. The injection of bvPLA2 was started 10–14 days after SNL surgery, and the injection was performed once a day and repeated twice. We found that the paw withdrawal threshold was significantly increased in SNL rats after two repetitive injections. Comparing the analgesic effect of bvPLA2 on SNL rats with other conventional treatment such as gabapentin [22,23], it is noteworthy that repetitive bvPLA2-mediated pain relief showed long-lasting characteristics, as measured by the paw withdrawal threshold of SNL rats 24 h after bvPLA2 treatment. In a previous study, it was shown that BV treatment induced analgesic effect that lasted more than several hours and prolonged morphine-mediated analgesia on the CIPN model [24]. This long-lasting cumulative effect makes bvPLA2 an attractive target for combined therapy with other treatments.

Many studies have reported that the analgesic effects of BV are mostly mediated by the adrenergic system [6,25,26,27,28]. Likewise, in our previous study, we showed that the analgesic effects of bvPLA2 on neuropathic pain induced by injection of oxaliplatin were mediated by the adrenergic system, especially via spinal α_2_-ARs [15]. In this regard, we investigated the mechanism of analgesic effects of bvPLA2 focusing on the noradrenergic pain modulation, expecting that the spinal α_2_-ARs would be involved. Interestingly, however, our results showed that the analgesic effect of bvPLA2 on SNL-induced neuropathic pain was mediated by α_1_-AR, not by α_2_-AR. Intrathecal treatment with α_1_-AR antagonist prazosin, but not the α_2_-AR antagonist idazoxan, prior to bvPLA2 treatment canceled out the analgesic effect of bvPLA2 on SNL-induced mechanical allodynia. This discrepancy might be due to the expressional change of the ARs in different conditions of neuropathic pain [29,30,31]. 

Previous studies have reported that the activation of spinal α_2_-ARs exerts antinociceptive effect [32], whereas the activation of spinal α_1_-AR evokes pronociceptive effect [33,34]. However, there have been a few studies reporting the potential analgesic effects of α_1_-AR activation. For example, one study reported that more pain behaviors were observed in α_1d_ AR-knockout mice compared with wild-type mice [35]. Another study showed that, in formalin-induced pain state, α1-AR agonists induced the antinociception [36]. Through electrophysiological methods, researchers showed that activation of α_1_-ARs suppressed the activity of spinal dorsal horn neurons through increased GABAergic inhibitory transmission [37,38]. To date, whether the effect of spinal α1-AR activation is antinociceptive or pronociceptive in nerve injury-induced neuropathic pain states has been uncertain. Therefore, we performed further experiments to check whether direct activation of α1-ARs could reduce the sustained mechanical allodynia after SNL. We found that the activation of spinal α1-ARs by intrathecal injection of phenylephrine ameliorated the mechanical allodynia, supporting the notion that spinal α1-AR activation evokes analgesic effect in SNL-induced neuropathic pain condition. 

**Figure 4:**
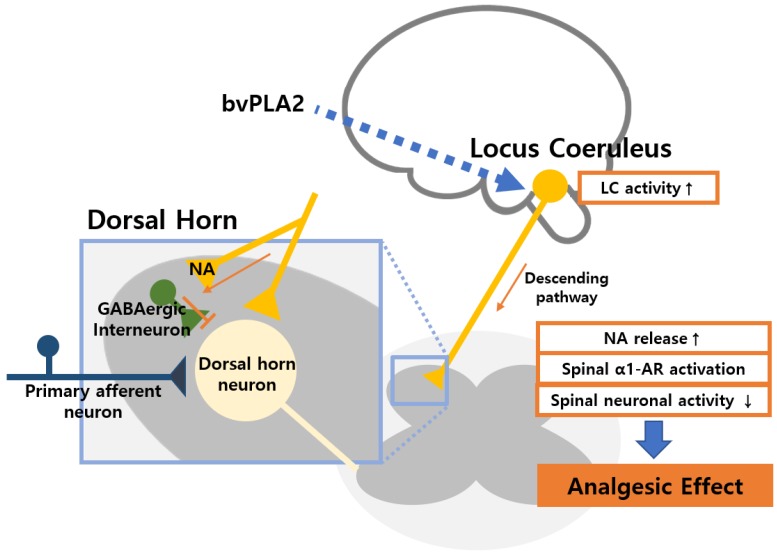
Schematics of the expected mechanism of bvPLA2-mediated analgesic effect. The bvPLA2 increases noradrenergic neurons in the locus coeruleus and facilitates noradrenaline (NA) release in the spinal cord. The α1-AR in GABAergic interneuron in the spinal cord dorsal horn is activated by the NA, which inhibits transmission of nociceptive signals.

Biochemical pathway through which the bvPLA2 induces activation of noradrenergic pain modulatory system is currently unknown in detail. In the case of BV, previous researchers have found that repetitive treatment with diluted BV increases activity of noradrenergic neurons in the locus coeruleus and suppresses phosphorylation of NR1 in the spinal cord [6]. Being the major component of BV, bvPLA2 might play a role in the activation of noradrenergic neurons in the locus coeruleus and concurrent modulation of spinal transmission (Figure 4). Previous studies of various pain models have demonstrated that treatment with BV or bvPLA2 induces analgesic effect, which is mediated by spinal AR activation [5,6,15,24]. Although spinal AR activation was involved in bvPLA2-mediated analgesic effect in this study, further studies are needed to clarify the interaction between bvPLA2, activation of noradrenergic neurons, and modulation of AR activation. Functional interaction between bvPLA2 injected externally and various isoforms of PLA2 produced endogenously should also be considered [39]. A long-term project would be needed to achieve this goal. Another well-known role of bvPLA2 is immune modulation [11]. Although bvPLA2 is known to be an allergen, the possible adverse side effect of allergic response could be managed by prior allergy tests and appropriate medical aid. Furthermore, recent studies have found that bvPLA2 suppresses allergic responses via regulation of Treg cells. The modulatory effect of bvPLA2 on Treg cells might be related to its analgesic effect on neuropathic pain [40,41]. According to previous studies, increasing Treg cells attenuate the neuropathic pain symptoms, and the depletion of Foxp3+ Treg cells promotes nerve injury-induced pain hypersensitivity [42,43]. In our previous study, we found that the preventive effects of bvPLA2 on oxaliplatin-induced neuropathic pain were mediated by its regulation of Treg cells [14]. Although we did not attempt to determine whether the analgesic effect of bvPLA2 on SNL-induced neuropathic pain is via this pathway to activate Treg cells and whether immunological changes induced by bvPLA2 affect the spinal AR, it would be of high interest to further investigate these mechanisms.

In conclusion, repetitive bvPLA2 (0.2 mg/kg/day, i.p.) injection for two days induced marked long-term improvement in the mechanical allodynia induced by SNL. The relieving effect of bvPLA2 was blocked by intrathecal injection of α1-AR antagonist prazosin but not by α2-AR antagonist idazoxan, indicating that the analgesic effect of bvPLA2 on SNL-induced neuropathic pain is mediated by spinal α1-ARs. Furthermore, we directly activated spinal α1-ARs using an intrathecal injection of α1-AR agonist phenylephrine and confirmed that α1-AR activation could inhibit the mechanical allodynia induced by nerve injury. 

Based on our result, we suppose that bvPLA2 could be an analgesic to treat neuropathic pain. As bvPLA2 has risk of anaphylaxis, a safer treatment approach will be required, such as combination with another drug to block any allergic response.

## 4. Materials and Methods

### 4.1. Animals

Adult male Sprague Dawley rats (Samtako, Seoul, Korea) were housed in cages with free food and water. The room was controlled to keep a constant temperature of 23 ± 2 °C and maintain a 12 h light/dark cycle. All procedures were approved on 19 February 2019 by the Institutional Animal Care and Use Committee at Kyung Hee University (KHUASP[SE]-19-012). Animals were randomly assigned to the surgery/treatment groups, and experimenters were blinded to the group throughout the experiments.

### 4.2. Spinal Nerve Ligation

Adult Sprague Dawley rats (8 weeks old) were randomly assigned to SNL or sham surgery. To induce neuropathic pain, right L5 SNL surgery was performed as previously described [21]. Under isoflurane anesthesia, the hair of the lower back was shaved, and a longitudinal skin incision was made on the right side of the spinal L6 level. After the elimination of L6 transverse process, the L5 nerve was carefully isolated and tightly ligated. Then, all procedures were completed by suturing the skin. In sham surgery, all the procedures were the same as the SNL surgery except for the L5 nerve ligation.

### 4.3. Behavioral Test 

To evaluate the development of allodynia and the analgesic effect of bvPLA2, von Frey test was performed. Before the test, all rats were acclimated for 30 min in a transparent acrylic box with a mesh floor. We applied mechanical stimuli with a series of von Frey filaments (Stoelting Co., Wood Dale, IL, USA) in the middle of the right hind paw and observed aversive response pattern (up-down method) [44,45]. PWT of 15 g was applied as the cut-off, and rats with a PWT less than 10 g at presurgery condition were excluded in surgery. 

### 4.4. bvPLA2 Administration

All treatments were carried out in rats at 10–16 days following SNL when the mechanical allodynia was fully developed. Phosphate-buffered saline (PBS) was used to dissolve bvPLA2 (Sigma, St. Louis, MO, USA), and bvPLA2 was injected intraperitoneally (0.2 mg/kg, i.p.) on two consecutive days [15]. We carefully determined the dose of bvPLA2 to evoke sufficient analgesic effect without toxicity or anaphylactic response based on previous studies [5,14,15,46,47]. The same volume of PBS was administrated to the control group. The von Frey test was performed prior to the administration of bvPLA2 and 24 h after the administration. 

### 4.5. Adrenergic Antagonist Administration

To investigate the mechanism of bvPLA2-induced analgesic effect, we made four groups: dimethyl sulfoxide (DMSO; Sigma, St. Louis, MO, USA) + bvPLA2, prazosin + bvPLA2, PBS + bvPLA2, and idazoxan + bvPLA2. DMSO 20% was used to dissolve α_1_-AR antagonist prazosin (Sigma, St. Louis, MO, USA; 30 μg). PBS was used to dissolve α_2_-AR antagonist idazoxan (Sigma, St. Louis, MO, USA; 50 μg). Under light isoflurane anesthesia, antagonist or vehicle solution was administered intrathecally to the SNL animals 20 min before bvPLA2 i.p. injection. Rats awoke from the anesthesia state within 5 min. The von Frey test was performed prior to the administration of bvPLA2 and 24 h after the administration. 

### 4.6. Adrenergic Agonist Administration

To investigate the effect of α_1_-AR agonist in SNL-induced mechanical allodynia, we intrathecally injected phenylephrine (Sigma, St. Louis, MO, USA; 50 μg) under light isoflurane anesthesia. Phenylephrine was dissolved in PBS. Rats awoke from the anesthesia state within 5 min. The von Frey test was performed prior to the administration of bvPLA2 and 1 h after the administration. 

### 4.7. Statistical Analysis

For statistical analysis and graphic works, we used Prism 7.0 (GraphPad Software, San Diego, CA, USA). The experimental data are presented as mean ± standard error of mean (SEM) and were analyzed by paired *t*-test, two-way ANOVA, followed by Bonferroni’s multiple comparison test to identify the statistical difference. *p* < 0.05 was considered significant.
